# Correction: Trotta et al. Sex and Age-Related Differences in Neuroinflammation and Apoptosis in *Balb/c* Mice Retina Involve Resolvin D1. *Int. J. Mol. Sci.* 2021, *22*, 6280

**DOI:** 10.3390/ijms27093956

**Published:** 2026-04-29

**Authors:** Maria Consiglia Trotta, Sami Gharbia, Hildegard Herman, Bianca Mladin, Andrei Hermenean, Cornel Balta, Coralia Cotoraci, Victor Eduard Peteu, Carlo Gesualdo, Francesco Petrillo, Marilena Galdiero, Roberto Alfano, Mihaela Gherghiceanu, Michele D’Amico, Settimio Rossi, Anca Hermenean

**Affiliations:** 1 Section of Pharmacology, Department of Experimental Medicine, University of Campania “Luigi Vanvitelli”, Via Santa Maria di Costantinopoli 16, 80138 Naples, Italy; mariaconsiglia.trotta2@unicampania.it (M.C.T.); marilena.galdiero@unicampania.it (M.G.); 2 “Aurel Ardelean” Institute of Life Sciences, Vasile Goldis Western University of Arad, 86 Revolutiei Av., 310144 Arad, Romania; gharbia.sami@uvvg.ro (S.G.); herman.hildegard@uvvg.ro (H.H.); mladin.bianca@uvvg.ro (B.M.); hermenean.anca@uvvg.ro (A.H.); 3 Faculty of Medicine, Carol Davila University of Medicine and Pharmacy, 8 Eroii Sanitari Av., 050474 Bucharest, Romaniamihaela.gherghiceanu@ivb.ro (M.G.); 4 Faculty of Medicine, Vasile Goldis Western University of Arad, 86 Revolutiei Av., 310144 Arad, Romania; cotoraci.coralia@uvvg.ro; 5 Victor Babes National Institute of Pathology, 99-101 Splaiul Independentei Av., 050096 Bucharest, Romania; peteuvictoreduard@gmail.com; 6 Eye Clinic, Multidisciplinary Department of Medical, Surgical and Dental Sciences, University of Campania “Luigi Vanvitelli”, Via Luigi De Crecchio 6, 80138 Naples, Italy; carlo.gesualdo@unicampania.it (C.G.); settimio.rossi@unicampania.it (S.R.); 7 Department of Ophthalmology, University of Catania, P.zza Università 2, 95131 Catania, Italy; francescopetrillo09@gmail.com; 8 Department of Advanced Medical and Surgical Sciences “DAMSS”, University of Campania “Luigi Vanvitelli”, P.zza L. Miraglia 2, 80138 Naples, Italy; roberto.alfano@unicampania.it

In the original publication, there was a mistake in Figure 4 as published [1]. In the initial version of the manuscript, the images for Figure 4A(b,c) were inadvertently mis-selected due to a technical oversight during figure assembly. This error has now been corrected, and the revised figure accurately represents the data used in the analysis. The corrected Figure 4 appears below. The authors state that the scientific conclusions are unaffected. This correction was approved by the Academic Editor. The original publication has also been updated.

## Figures and Tables

**Figure 4:**
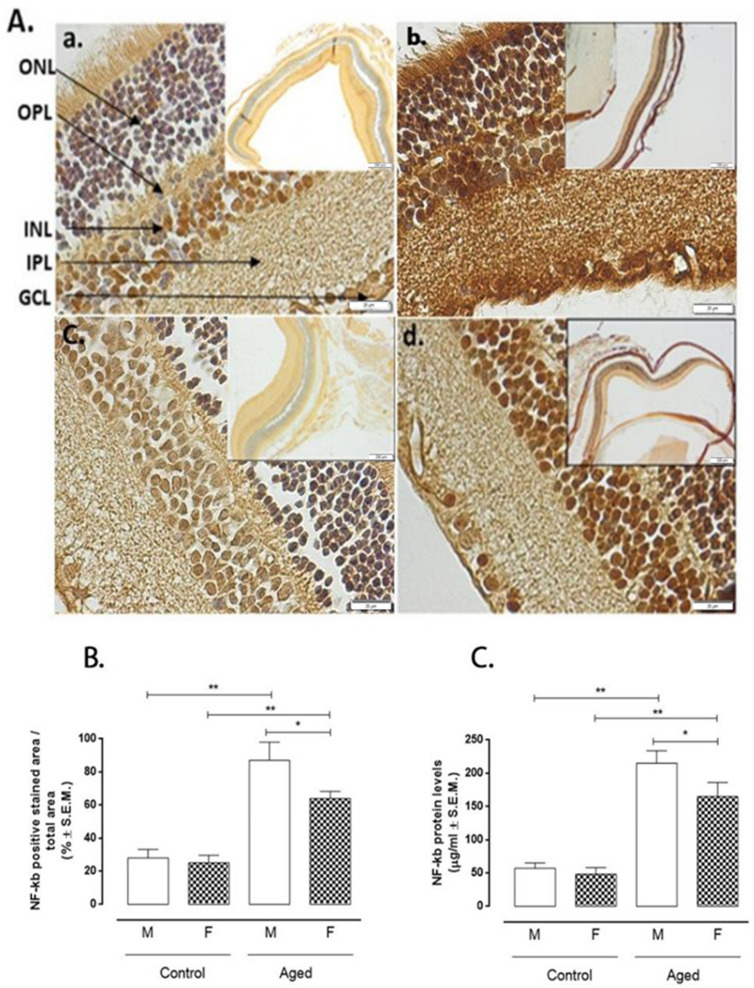
NF-kB expression in aged and younger (control) retina. (**A**) Representative immunohistochemistry (a. control male; b. aged male; c. control female; d. aged female) and (**B**) quantification of immunopositive areas in control and aged retina (M: males and F: females); the results calculated as the % ± S.E.M. are considered statistically significant when * *p* < 0.05; ** *p* < 0.01; *n* = 10 observations for each individual/group; ONL: outer nuclear layer; OPL: outer plexiform layer; INL: inner nuclear layer; IPL: inner plexiform layer; GCL: ganglion cell layer; Panel A: bar = 20 µm. The frames in panel A are given on the right in a lower magnification bar = 200 µm; (**C**) enzyme-linked immunosorbent assay (ELISA) of NF-kB protein levels in the control and aged retina; results are expressed as the mean ± S.E.M. of *n* = 10 retinas per group. DU: densitometric units; * *p* < 0.05; ** *p* < 0.01.
